# Mid- and long-term results of primary vs. secondary total elbow arthroplasty after index intra-articular fracture of the distal humerus in the elderly

**DOI:** 10.1016/j.jsea.2026.100027

**Published:** 2026-04-29

**Authors:** Oliver Deml, Philipp Schenk, Clemens Fritsche, Philipp Kobbe, Thomas Mendel, Jörg Eschweiler, Christian Fischer

**Affiliations:** a Department of Trauma and Reconstructive Surgery, BG Klinikum Bergmannstrost Halle gGmbH, Halle, Saale, Germany; b Innovation Hub Musculoskeletal Surgery Halle, Surgery, BG Klinikum Bergmannstrost Halle gGmbH, Halle, Saale, Germany; c Department of Trauma, Hand and Reconstructive Surgery, Martin Luther University Hospital Halle, Halle, Saale, Germany

**Keywords:** Distal humerus fracture, Total elbow arthroplasty, Primary vs. secondary TEA, Geriatric patients, Upper extremity trauma

## Abstract

**Background:**

Geriatric distal humerus fractures are difficult to manage. Open reduction and internal fixation remain the standard, but total elbow arthroplasty (TEA) appears to be a viable alternative. This study evaluated the long-term outcome after primary TEA in comparison to secondary TEA.

**Methods:**

In this retrospective cohort study with prospective follow-up (FU), patients undergoing TEA after complex distal humerus fractures were analyzed as either primary TEA (index treatment; n = 21) or secondary TEA (salvage procedure after failed fracture treatment; n = 13). Data extracted from the medical records and the final clinical FU included demographics, fracture type, Mayo Elbow Performance Score, Disabilities of the Arm, Shoulder and Hand, patient satisfaction, range of motion (flexion–extension and pronation–supination), normalized handgrip strength, Janda muscle strength grades (flexion, extension, pronation, supination), complications, and revisions.

**Results:**

From 2003 to 2012, 57 TEAs were performed; 34 patients with semiconstrained prosthesis models met the study criteria and were available for analysis (21 primary, 13 secondary; 7 men, 27 women). The remaining 23 cases were excluded because of non–post-traumatic indications, prosthesis types outside the inclusion criteria, or unavailable analyzable FU of at least 5 years. Mean FU was 8 years (range, 5-13). Mean age at surgery was 62 ± 15 years (23-85), with no difference between primary and secondary TEA (*P* = 1.000). Primary TEA was associated with a higher Mayo Elbow Performance Score than secondary TEA (87 ± 11 vs. 74 ± 11; *P* = .002), a lower Disabilities of the Arm, Shoulder and Hand score (28 ± 17 vs. 39 ± 10; *P* = .042), higher patient satisfaction (*P* = .009), and better extension strength (*P* = .022). Mean normalized handgrip strength was higher after primary TEA (0.75 ± 0.21 vs. 0.58 ± 0.27), but this difference narrowly missed statistical significance (*P* = .050). Mean flexion–extension arc was 102° ± 24° vs. 88° ± 33° (*P* = .212), and mean pronation–supination arc was 155° ± 28° vs. 150° ± 22° (*P* = .214). Complications were numerically less frequent after primary TEA (43% vs. 69%; *P* = .172), as were revisions (18% vs. 39%; *P* = .211), but these differences were not statistically significant.

**Conclusion:**

In this Level III retrospective cohort comparison, primary TEA was associated with better mid- to long-term functional scores and patient satisfaction than secondary TEA. However, several clinically relevant numerical differences, including range of motion, complication rate, and revision rate, did not reach statistical significance and should be interpreted cautiously in light of the limited sample size, retrospective design, and potential selection bias. Prospective multicenter studies and registry data are needed to refine indications and long-term expectations.

Distal humerus fractures in older adults are difficult to reconstruct owing to osteoporotic bone, comminution, and comorbidity. Although open reduction and internal fixation (ORIF) remains the standard, contemporary evidence supports total elbow arthroplasty (TEA) as an effective procedure for nonreconstructible fractures in the geriatric population, offering reliable pain relief and favorable function.^2^^,^^8^ Across comparative studies, TEA tends to yield comparable or better post-operative elbow motion than ORIF, particularly in elderly patients with complex distal humerus fractures.^14^^,^^15^ Reoperation and post-operative stiffness rates are generally lower after TEA, whereas Mayo Elbow Performance Score (MEPS) and Disabilities of the Arm, Shoulder and Hand (DASH) scores do not differ significantly between TEA and ORIF, suggesting similar overall functional outcomes.^1^^,^^6^^,^^16^

Despite these, TEA carries nontrivial risks. Across systematic reviews of primary TEA for acute distal humerus fractures, overall complication rates of roughly 20%-35% are reported; common events include ulnar neuropathy, periprosthetic fracture, heterotopic ossification, polyethylene wear, and stem loosening.^11^^,^^12^^,^^21^ On the other hand, short-term follow-up (FU) of similar studies suggests durable implant survival with low revision requirements in elderly fracture patients, but vigilance for mechanical and biological failure remains essential.^1^^,^^6^^,^^14^^,^^16^

Primary TEA is associated with better functional outcomes and less pain than secondary TEA in the short- to midterm, whereas delayed implantation, particularly after failed nonoperative treatment or fixation, has been linked to inferior functional outcome. Salvage TEA shows higher rates of revision, periprosthetic fracture, infection, and wound complications, and although earlier conversion after failed fixation may mitigate these risks, it does not abolish them.^11^^,^^12^^,^^15^^,^^21^ Overall, these data support preferential consideration of primary TEA in carefully selected geriatric patients with nonreconstructible fracture patterns after short-term FU, as secondary TEA is associated with higher complication rates and worse function.

Therefore, we conducted a comparative cohort study of primary vs. secondary TEA for distal humerus fractures to investigate whether these differences persist at long-term FU, assessing long-term clinical function, patient-reported outcomes, and adverse events.

## Materials and methods

This retrospective cohort study with prospective FU was conducted following approval by the responsible ethics committee and in compliance with the ethical principles of the Declaration of Helsinki for medical research in its most recent form (positive vote no. 2025-242). All patients provided informed consent before study participation and data collection. The study included patients with acute distal humerus fractures treated with primary TEA and patients undergoing secondary TEA as a salvage procedure after failed fracture treatment, with a minimum FU of ≥5 years. The source population comprised 57 TEAs performed between 2003 and 2012. Patients' age, sex, fracture classification, prosthesis model, date of surgery, inpatient stay, and timing of the last FU examination were extracted from the medical records. Because of the retrospective design, baseline functional scores were not consistently available and were therefore not analyzed. With regard to the primary question, the overall cohort was divided into:

• Primary TEA: index treatment following distal humerus fracture
• Secondary TEA: conversion arthroplasty after failed fracture treatment (eg, failed osteosynthesis and post-traumatic sequelae such as malunion/nonunion or post-traumatic osteoarthritis)

Patients with rheumatoid arthritis, primary osteoarthritis, or tumor-related pathology were not included. In addition, cases with prosthesis types outside the predefined semiconstrained implants and cases without analyzable FU of at least 5 years were not entered into the final analysis. Consequently, 34 patients (21 primary TEA, 13 secondary TEA; 7 men, 27 women; mean age 62 ± 15 years, range 23-85) were included. Patients were treated with semiconstrained prosthesis models (n = 30 Discovery, Biomet, Warsaw, Indiana, United States; n = 4 Coonrad–Morrey, Zimmer, Warsaw, Indiana, United States).

The duration of the inpatient stay was recorded from the date of surgery until hospital discharge. During the study period, these procedures were routinely performed on an inpatient basis at our institution because of the age and frailty of many patients, the need for post-operative pain control and wound monitoring, and supervised initiation of rehabilitation. Fractures were classified according to the established AO Foundation (Arbeitsgemeinschaft für Osteosynthesefragen) classification for fractures and dislocations by 2 experienced surgeons.

Data were obtained from the medical records and from the last available prospective clinical FU visit. FU intervals were not fully standardized over the long inclusion period; for the present analysis, the most recent examination at a minimum of 5 years after TEA was used. Arm strength, mobility, functional outcome, and patient satisfaction were assessed at this final FU. A standardized radiographic outcome analysis was not available for all patients and was therefore not included as a formal study endpoint.

Elbow strength was assessed according to the Janda muscle strength grading system in flexion, extension, supination, and pronation. Janda grades, handgrip strength, and range of motion (ROM) were assessed by 2 experienced examiners at the final FU. Examiner blinding was not feasible because of the retrospective design and the obvious surgical history. Strength of the contralateral side was measured in the same way. Handgrip strength of the affected and healthy limb was measured using a handgrip dynamometer (Seahan Industries, Eschborn, Germany). Normalized handgrip strength was calculated as strength on the prosthesis side divided by strength on the contralateral side. ROM was assessed clinically using goniometric examination for flexion, extension, pronation, and supination and summarized as the flexion–extension and pronation–supination arcs.

Elbow-specific function outcome and disability were evaluated using the DASH score, which assesses pain-related impairment and limitations in upper limb activities, and the MEPS, which incorporates pain, ROM, stability, and daily function. Both instruments are internationally validated outcome measures, and their scoring algorithms are not detailed here.^3^^,^^5^^,^^7^

Patient satisfaction was recorded using a numeric rating scale (0 = dissatisfied, 10 = very satisfied).

Complications were assessed from chart review and FU examination and categorized as early (<6 weeks) or late (≥6 weeks) post-operative events. Complications requiring unplanned reoperation or component revision were considered major complications.

### Statistics

Differences between patients with primary or secondary prosthesis in sex and fracture morphology according to the AO Foundation classification were assessed using cross-tabulation and Fisher exact test. Independent-samples *t* tests were used to compare age at surgery, age at FU, hospital stay, and FU duration. Differences in patients' strength and mobility were analyzed using separate repeated-measures general linear models (GLM) (GLM for repeated measures), with movement direction (flexion, extension, pronation, supination) as the within-subject factor and treatment group as the between-group factor. Bonferroni-adjusted post hoc testing was used for pairwise comparisons. Separate univariate GLMs were used for normalized handgrip strength, MEPS, DASH, and patient satisfaction. Janda grades and ROM variables were correlated with MEPS, DASH, and patient satisfaction using Spearman or Pearson correlation analyses, as appropriate. Effect sizes (Cohen d) were calculated for key between-group differences. Kaplan–Meier survival analysis and the log-rank test were used to compare implant survival. Because of the retrospective design and fixed sample size, no a priori power analysis was performed; therefore, nonsignificant between-group differences were interpreted cautiously and in conjunction with effect sizes and absolute group differences. Statistical significance was defined as *P* < .05. Analyses were performed using IBM SPSS Statistics, version 29 (IBM Corp., Armonk, NY, USA).

## Results

Of the 57 TEAs performed during the study period, 34 patients met the inclusion criteria and completed the final analysis: 21 primary TEAs and 13 secondary TEAs. No differences in age at the time of surgery (*P* = .124) or at the time of final FU (*P* = .197) were found. No differences between the primary and secondary prosthesis groups were found for the type of prosthesis (*P* = 1.000) or the injured side (*P* = .465). The patients with primary prosthesis showed C3-type fractures in 86% of cases (n = 18). Patients in the group with secondary prosthesis showed a more heterogeneous fracture distribution, with C3-type fractures in 23% of cases. The fracture type distribution differed between groups (Table I, *P* = .002), reflecting the nonrandomized treatment selection and the potential for selection bias. In one patient with a C1 fracture, primary implantation was performed in agreement with the patient to reduce the risk of further operations because of blindness and the associated individual circumstances.

**Table I:** Frequency of fracture morphology for primary and secondary TEA.

AO Foundation type	Fracture	Primary TEA	Secondary TEA	Total
	A2	-	1 (8%)	1 (3%)
	A3	-	2 (15%)	2 (6%)
	B3	-	1 (8%)	1 (3%)
	C1	1 (5%)	4 (31%)	5 (15%)
	C2	2 (10%)	1 (8%)	3 (9%)
	C3	18 (86%)	3 (23%)	21 (62%)
	Other	-	1 (8%)	1 (3%)
Total		21 (100%)	13 (100%)	34 (100%)

*TEA*, total elbow arthroplasty.

The mean hospital stay was 14 ± 5 days overall (range: 7-30 days) and did not differ significantly between groups (*P* = .082). All procedures were managed as inpatient cases during the study period. In one case (a polytraumatized patient with severe comorbidities and primary TEA), the hospital stay was 74 days because of the associated injuries; this outlier was excluded from the between-group comparison of hospital stay. The FU period was 8 ± 2 years and did not differ between groups (*P* = .240), ranging from 5 years (5 cases) to 13 years (3 cases). In 27% (n = 9) of the patients, their final FU was seven years after surgery.

### Patients' strength

**Figure 1:**
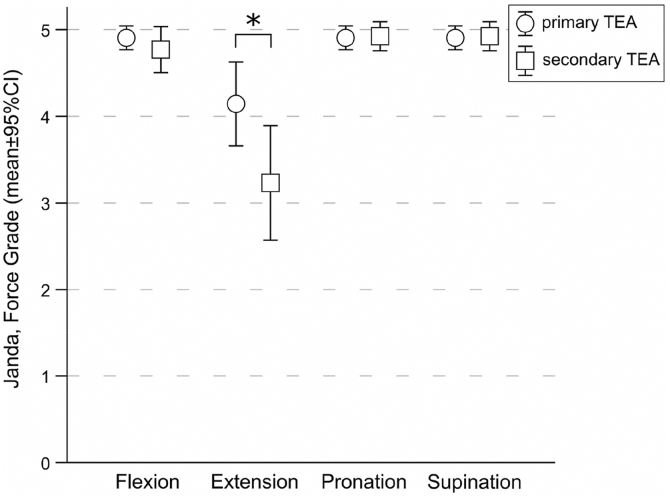
Janda force grade in 4 directions for patients with primary and secondary total elbow arthroplasty. ∗ indicates a statistically significant difference between groups for extension (*P* = .022). *TEA*, total elbow arthroplasty; *CI*, confidence interval.

Figure 1 shows the measured Janda force grade for the 4 directions and both patient groups. The post hoc pairwise comparisons for flexion (*P* = .292), pronation (*P* = .860), and supination (*P* = .860) showed no significant differences between the groups. In extension, the patients with primary prosthesis showed higher forces with 1 ± 1 force grade to Janda, compared to patients with secondary prosthesis (*P* = .022). In the primary prosthesis group, grade 5 in Janda was reached in 90% (n = 19) for flexion, 48% (n = 10) for extension, and each 90% (n = 19) for pronation and supination, respectively. In patients with secondary prosthesis, grade 5 according to Janda was achieved in 77% (n = 10) for flexion, 8% (n = 1) for extension, and 92% (n = 12) each for pronation and supination.

No differences could be found for the uninjured side, nor between the directions (*P* = 1.000) or between the groups (*P* = .292), respectively.

### Range of motion

**Figure 2:**
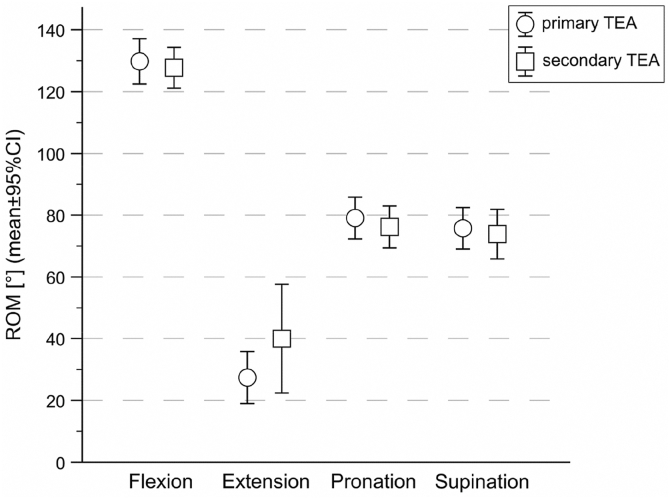
Range of motion in 4 directions for patients with primary and secondary total elbow arthroplasty. *TEA*, total elbow arthroplasty; *CI*, confidence interval; *ROM*, range of motion.

ROM differed significantly between movement directions (flexion–extension vs. pronation–supination; *P* < .001) (Fig. 2). However, no significant overall group effect was found between the primary and secondary TEA groups, and no significant interaction between group and direction was detected (*P* = .178). Mean flexion–extension ROM was 102° ± 24° in the primary TEA group vs. 88° ± 33° in the secondary TEA group (mean difference, 14°; *P* = .212), and mean pronation–supination ROM was 155° ± 28° vs. 150° ± 22°, respectively (mean difference, 5°; *P* = .214) (Fig. 2). These numerical differences did not reach statistical significance.

### Hand grip strength

**Figure 3:**
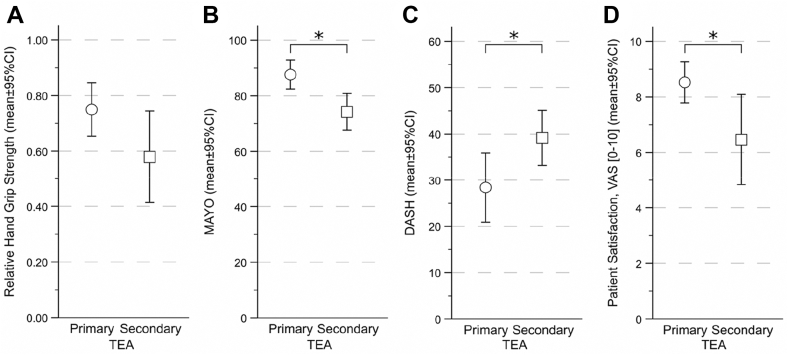
Functional outcome and patient satisfaction of patients with primary and secondary total elbow arthroplasty. ∗ indicates statistically significant differences between groups for MEPS (*P* = .002), DASH (*P* = .042), patient satisfaction (*P* = .009), and normalized handgrip strength (*P* = .050). *VAS*, visual analog scale; *TEA*, total elbow arthroplasty; *CI*, confidence interval; *MEPS*, Mayo Elbow Performance Score; *DASH*, Disabilities of the Arm, Shoulder and Hand.

Patients with primary prosthesis showed a mean normalized handgrip strength of 0.75 ± 0.21, compared with 0.58 ± 0.27 in patients with secondary prosthesis (*P* = .050, Fig. 3). The effect size (Cohen d = 0.74; 95% confidence interval [CI], 0.01-1.44) indicates a moderate-to-large difference despite the borderline *P* value.

### Functional outcome

The GLM showed significantly better functional scores in the primary prosthesis group for both MEPS (*P* = .002) and DASH (*P* = .042). The effect size for the MEPS score was large (Cohen d = 1.18; 95% CI, 0.43-1.93). For the patients with primary prosthesis, the mean MEPS was 87 ± 11, whereas the mean MEPS was 74 ± 11 for the patients with secondary prosthesis. The mean DASH score was 28 ± 17 after primary TEA and 39 ± 10 after secondary TEA, with Cohen d = 1.21 (95% CI, 0.46-1.97). Spearman correlation showed a significant association only between Janda extension strength and both the MEPS (*P* = .031, r = 0.370) and DASH scores (*P* = .001, r = −0.534). For Janda grade in the other directions, no significant correlations were found for MEPS or DASH scores (flexion *P* > .060, pronation *P* > .108, supination *P* > .108).

Regarding the correlation of the ROM with MEPS and DASH scores, only for the ROM in flexion, a significant correlation was found with the MEPS score (r = 0.477, *P* = .004). All other pairwise correlations showed no significance with *P* > .122.

### Patient satisfaction

Patient-reported satisfaction on the 0-10 numeric rating scale (10 = excellent, scores ≥8 considered good satisfaction) was significantly higher in the primary TEA group (8.5 ± 1.6) than in the secondary TEA group (6.5 ± 2.7; *P* = .009), with Cohen d = 0.96 (95% CI, 0.23-1.69). Patient satisfaction correlated significantly with the MEPS score (r = 0.578, *P* < .001), whereas no significant correlations were found with Janda muscle strength grades in any direction, ROM in any direction, or the DASH score (all *P* > .054).

### Complications

A total of 53% of all patients showed at least one complication. The overall complication rate was numerically lower after primary than after secondary TEA (43% vs. 69%), but this difference did not reach statistical significance (*P* = .172). Early post-operative complications (<6 weeks) occurred in 27% of patients overall (19% vs. 39%, *P* = .254). Late complications (≥6 weeks) occurred in 32% of all patients (24% vs. 46%, *P* = .262). Mechanical failure of the coupling module occurred exclusively in secondary TEA in 4 cases and was therefore more frequent than in primary TEA (*P* = .015). However, no significant difference in the rate of coupling mechanism failure was observed between the 2 prosthesis models. Although several complication comparisons were not statistically significant, the absolute between-group differences should be interpreted cautiously because the study was not powered for these endpoints.

Revision surgery, representing major complications, was required in 18% of patients in the primary group and 39% in the secondary group (*P* = .211). In primary TEA, revisions were mostly performed for ankylosis or periprosthetic fracture. In contrast, revisions in secondary TEA were predominantly due to mechanical failure of the coupling module. A detailed overview of the recorded complications, including frequencies per group and statistical comparisons, is presented in Table II.

**Table II:** Summary of early and late complications, implant failures, and revision procedures in patients with primary and secondary total elbow arthroplasty (TEA), including overall incidence and statistical comparison between groups.

Complications	Primary TEA (*n* = 21)	Secondary TEA (*n* = 13)	Total	*P* value
Total complications	9 (42.9%)	9 (69.2%)	18 (52.9%)	.172
Early post-operative complications (<6 weeks)	4 (19%)	5 (38.5%)	9 (26.5%)	.254
Hematoma with tension blisters	1 (4.8%)	1 (7.7%)	2 (5.9%)	
Post-operative flush/swelling	0	2 (15.4%)	2 (5.9%)	
Wound seroma	0	2 (15.4%)	2 (5.9%)	
Periprosthetic ulnar shaft fracture	1 (4.8%)	0	1 (2.9%)	
New ulnar nerve hypesthesia	1 (4.8%)	0	1 (2.9%)	
Respiratory insufficiency	1 (4.8%)	0	1 (2%)	
Late complications (>6 weeks)	5 (23.8%)	6 (46.2%)	11 (32.4%)	.262
Paresthesia	2 (9.5%)	2 (15.4%)	4 (11.8%)	
Progressive hypesthesia	0	1 (7.7%)	1 (2.9%)	
Post-traumatic humeral head fracture	0	1 (7.7%)	1 (2.9%)	
Ipsilateral wrist pain (denervation)	0	1 (7.7%)	1 (2.9%)	
Flexion contracture with triceps tendon rupture	0	1 (7.7%)	1 (2.9%)	
Chronic bursitis	0	1 (7.7%)	1 (2.9%)	
Elbow stiffness	2 (9.5%)	0	2 (5.9%)	
Shoulder dislocation with stiffness	1 (4.8%)	0	1 (2.9%)	
Coupling module defect	0	4 (30.8%)	4 (11.8%)	.015
Revision surgery	3 (14.3%)	5 (38.5%)	8 (23.5%)	.211

**Figure 4:**
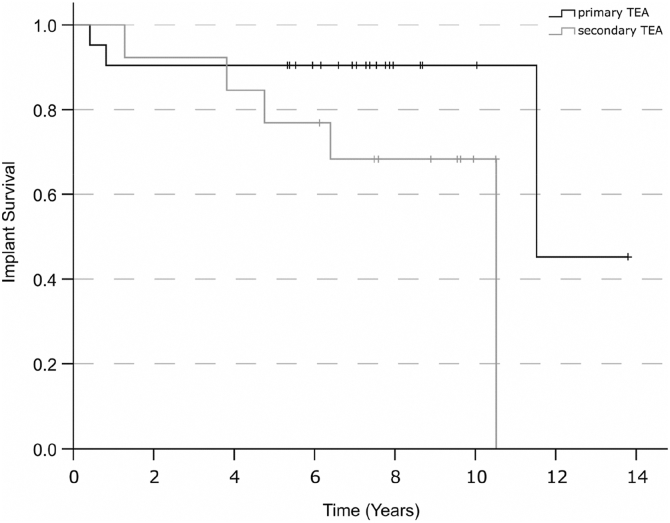
Kaplan–Meier survival curves comparing implant survival between patients with primary and secondary total elbow arthroplasty. *TEA*, total elbow arthroplasty.

The Kaplan–Meier analysis showed no differences in the time of implant survival between the 2 prosthesis groups (*P* = .068, Fig. 4).

## Discussion

In this Level III retrospective cohort comparison, primary TEA was associated with better mid- to long-term functional outcomes and higher patient satisfaction than secondary (salvage) TEA for complex distal humerus fractures in elderly patients. The most robust between-group differences were observed for MEPS, DASH, satisfaction, and extension strength. By contrast, differences in ROM, handgrip strength, complications, and revision surgery were directionally in favor of primary TEA but did not reach statistical significance. Accordingly, our findings suggest an advantage of primary TEA in this cohort, but they should be interpreted cautiously and not as definitive evidence of superiority.

The age and sex distribution in our cohort, characterized by predominantly older women and comparatively younger men, is in line with the reported epidemiology of distal humerus fractures.^11^^,^^12^^,^^15^^,^^18^^,^^21^ Recent comparative studies of primary vs. secondary TEA, including cohorts of approximately 22-33 primary and 17-66 secondary cases with 2-6 years of FU, likewise demonstrate superior or at least equivalent functional outcomes after primary TEA, with higher MEPS, lower DASH, greater ROM, and lower complication and reoperation rates. By contrast, secondary or revision TEA, particularly following failed nonoperative treatment or ORIF, is associated with inferior objective function and higher rates of mechanical and infectious complications, despite comparable pain levels and patient satisfaction. Our findings are concordant with these data and extend the available FU duration, although unlike prospective or registry-based analyses, our retrospective cohort remains susceptible to confounding by indication. This cautious interpretation is also consistent with registry and systematic-review data, which support acceptable function and implant survival after fracture-related TEA but emphasize the trade-off of non-negligible complication and revision risk over time.

In our cohort, both primary and secondary TEA restored a functional arc of motion in flexion–extension and forearm rotation sufficient for most activities of daily living, confirming that TEA effectively re-establishes basic elbow mobility.^11^^,^^12^^,^^16^^,^^21^ The 14° difference in flexion–extension arc favored primary TEA but did not reach statistical significance, likely in part because of the limited sample size. Secondary TEA, however, was associated with more pronounced strength deficits and inferior functional scores. At a mean FU of approximately 8 years, patients treated with primary TEA achieved MEPS predominantly in the “good–excellent” range and significantly lower (better) DASH scores than those after secondary TEA, indicating less disability and superior long-term function. These findings support previous short-term reports regarding better functional outcome of primary TEA than secondary TEA.

Complications and reoperations remain a central concern in post-traumatic elbow arthroplasty.^2^^,^^8^^,^^9^^,^^15^^,^^19^^,^^22^^,^^27^ The overall complication rate in our series was 53%, higher than typically reported for primary TEA in acute distal humerus fractures, which is at least partly attributable to the inclusion of complex secondary cases. Patients undergoing secondary TEA had higher complication rates (69% vs. 43%) and more frequent revision procedures (39% vs. 18%) than those treated with primary TEA, a clinically relevant gradient despite the absence of statistical significance. This is particularly relevant because the study was likely underpowered for complication-related endpoints. In comparison, ORIF, although still regarded as the standard treatment for many distal humerus fractures, is likewise associated with a substantial complication burden. Large meta-analyses report overall complication rates of roughly 53% and reoperation rates of about 21% after ORIF of intra-articular distal humerus fractures, with ulnar neuropathy, heterotopic ossification, stiffness, infection, implant failure, and nonunion among the most frequent adverse events. Functional outcomes are often favorable despite these problems, with mean arcs of motion of approximately 110°-125° and predominantly good to excellent standardized scores; however, up to 46% of patients fail to achieve a functional ROM, and around 24% develop post-traumatic osteoarthritis. Complication rates are particularly elevated in elderly patients and in complex fracture patterns, and the quality of fixation has a decisive impact on outcome. ORIF therefore remains a reliable option when stable fixation and early mobilization can be ensured, but its limitations in osteoporotic, highly comminuted fractures support the rationale for considering primary TEA in selected geriatric cases.

In the group with secondary TEA, many revisions were related to polyethylene wear, loosening, or periprosthetic fractures, underscoring the cumulative effect of multiple previous procedures and higher mechanical demands on implant survival. Prior studies have similarly shown that younger, more active patients are more susceptible to prosthetic wear and failure.^4^^,^^5^^,^^10^^,^^26^ With respect to polyethylene wear and coupling module defects, earlier work has demonstrated high revision rates in secondary TEA, attributed to increased mechanical loading and material properties.^4^^,^^17^^,^^26^ In our cohort, however, mechanical complication rates did not differ significantly according to patient age or prosthesis model. Design modifications such as enlarging contact surfaces to reduce peak stresses and the use of more wear-resistant polyethylene types are recommended strategies to mitigate these problems.^13^^,^^24^^,^^25^

Despite the relatively high complication rates and permanent activity restrictions, overall patient satisfaction was high, particularly after primary TEA.^18^^,^^25^ Patients in the primary group more frequently reported being satisfied or very satisfied with their elbow function, whereas satisfaction in the secondary group was more heterogeneous. This is likely related to the more favorable clinical course after primary TEA, with fewer procedures and more predictable pain relief and functional recovery, in contrast to the physical and psychological burden of failed fixation and subsequent salvage surgery. Both groups nevertheless showed clear improvement compared with their presumed pre-operative status, and some patients with residual deficits remained highly satisfied if pain was adequately controlled and independence in daily activities were preserved. Accordingly, objective scores and clinical findings should be interpreted in conjunction with patient-reported outcomes in the context of shared decision-making. Careful indication, thorough patient counseling, and strict consideration of compliance and activity restrictions are essential.

These results have direct implications for surgical strategy.^9^^,^^11^^,^^12^^,^^20^^,^^22^^,^^23^^,^^26^ ORIF remains the treatment of choice in younger patients and in fractures amenable to stable reconstruction, but in osteoporotic bone and highly comminuted intra-articular fractures the risk of fixation failure is substantial. Our data, together with the existing literature, support considering primary TEA as one possible option in selected geriatric patients with nonreconstructible distal humerus fractures. Relevant decision parameters include age, biological fitness, bone quality, fracture morphology, baseline functional requirements, and comorbidities. Primary TEA should not be viewed as a universal solution, but as an alternative for clearly defined indications in which fixation is unlikely to succeed and delayed arthroplasty could entail a higher complication burden. There is a need for structured treatment algorithms and indication criteria to identify those patients in whom an acute TEA as a first-line procedure may offer the greatest benefit.

Several limitations must be acknowledged. First, this is a Level III retrospective cohort comparison, and allocation to primary vs. secondary TEA was influenced by surgeon judgment, fracture pattern, prior treatment history, and patient-related factors, introducing substantial selection bias. This is reflected by the unequal fracture distribution between groups, with more C3 fractures in the primary TEA group and a heterogeneous secondary cohort after different failure scenarios. Second, the sample size of 34 patients, although relevant for this uncommon indication, limits statistical power; therefore, several clinically meaningful numerical differences did not reach statistical significance. No a priori power analysis was possible because of the retrospective design. Third, baseline patient-reported outcome measures, comorbidities, smoking status, obesity, and the exact interval from trauma to secondary TEA were not consistently available from the historical records. Likewise, the initial management pathways within the secondary group and detailed documentation on staged procedures were incomplete. Fourth, elbow strength was assessed with the semiquantitative Janda scale rather than dynamometry, and no standardized radiographic outcome assessment was available for all patients. Finally, the single-center setting and performance by experienced surgeons may limit generalizability. These limitations prevent robust causal conclusions and underscore that the present findings should be viewed as hypothesis-generating.

## Conclusion

This Level III retrospective cohort study suggests that primary TEA may be associated with better long-term functional scores and patient satisfaction than secondary TEA after failed fracture treatment.

However, conclusions must remain tempered because of the retrospective design, possible selection bias, and limited sample size. Numerical differences in ROM, complications, and revisions favored primary TEA but did not reach statistical significance. These findings may help inform treatment discussions in selected elderly patients with complex distal humerus fractures, but prospective multicenter studies and registry analyses are needed before stronger recommendations can be made.

## Disclaimers:

Funding: No funding was disclosed by the authors.

Conflicts of interest: The author, their immediate family, and any research foundation with which they are affiliated have not received any financial payments or other benefits from any commercial entity related to the subject of this article.

## References

[1] Al-Hamdani A., Rasmussen J.V., Holtz K., Olsen B.S. (2020). Elbow hemiarthroplasty versus open reduction and internal fixation for AO/OTA type 13 C2 and C3 fractures of distal humerus in patients aged 50 years or above: a randomized controlled trial. Trials.

[2] Al-Hamdani A., Rasmussen J.V., Olsen B.S. (2022). Good functional outcomes after open reduction and internal fixation for AO/OTA type 13-C2 and -C3 acute distal humeral fractures in patients aged over 45 years. J Shoulder Elbow Surg.

[3] Cusick M.C., Bonnaig N.S., Azar F.M., Mauck B.M., Smith R.A., Throckmorton T.W. (2014). Accuracy and reliability of the Mayo elbow performance score. J Hand Surg Am.

[4] Davey M.S., Hurley E.T., Gaafar M., Molony D., Mullett H., Pauzenberger L. (2021). Long-term outcomes of total elbow arthroplasty: a systematic review of studies at 10-year follow-up. J Shoulder Elbow Surg.

[5] Geurts E.J., Viveen J., van Riet R.P., Kodde I.F., Eygendaal D. (2019). Outcomes after revision total elbow arthroplasty: a systematic review. J Shoulder Elbow Surg.

[6] Githens M., Yao J., Sox A.H.S., Bishop J. (2014). Open reduction and internal fixation versus total elbow arthroplasty for the treatment of geriatric distal humerus fractures. J Orthop Trauma.

[7] Hudak P.L., Amadio P.C., Bombardier C., Beaton D., Cole D., Davis A. (1996). Development of an upper extremity outcome measure: the DASH (disabilities of the arm, shoulder, and head). Am J Ind Med.

[8] Jagadish U., Kumar K.V., Shanthappa A.H. (2023). Functional outcome of distal humerus fractures treated with open reduction and internal fixation with bicolumnar plating in a tertiary care setting. Cureus.

[9] Korner J., Lill H., Müller L.P., Hessmann M., Kopf K., Goldhahn J. (2005). Distal humerus fractures in elderly patients: results after open reduction and internal fixation. Osteoporos Int.

[10] Lee B.P., Adams R.A., Morrey B.F. (2005). Polyethylene wear Aeter total elbow arthroplasty [Internet]. http://www.Jbjs.ofg.

[11] Liu C., Zhang D., Blazar P., Earp B.E. (2023). Outcomes after Acute versus delayed total elbow arthroplasty for the treatment of distal humerus fractures. J Hand Surg Glob Online.

[12] Logli A.L., Shannon S.F., Boe C.C., Morrey M.E., O’Driscoll S.W., Sanchez-Sotelo J. (2020). Total elbow arthroplasty for distal humerus fractures provided similar outcomes when performed as a primary procedure or after failed internal fixation. J Orthop Trauma.

[13] Macken A.A., Prkic A., Kodde I.F., Lans J., Chen N.C., Eygendaal D. (2020). Global trends in indications for total elbow arthroplasty: a systematic review of national registries. EFORT Open Rev.

[14] McKee M.D., Veillette C.J.H., Hall J.A., Schemitsch E.H., Wild L.M., McCormack R. (2009). A multicenter, prospective, randomized, controlled trial of open reduction—internal fixation versus total elbow arthroplasty for displaced intra-articular distal humeral fractures in elderly patients. J Shoulder Elbow Surg.

[15] Palladino S., Baldairon F., Godet J., Clavert P. (2024). Outcomes of total elbow arthroplasty in the treatment of distal humeral fractures in the elderly: a retrospective cohort comparison between primary arthroplasty and arthroplasty secondary to failed internal fixation. J. Shoulder Elbow Surg.

[16] Prasad N., Dent C. (2008). Outcome of total elbow replacement for distal humeral fractures in the elderly. J Bone Joint Surg Br.

[17] Prkic A., Welsink C., The B., van den Bekerom M.P.J., Eygendaal D. (2017). Why does total elbow arthroplasty fail today? A systematic review of recent literature. Arch Orthop Trauma Surg.

[18] Ryu S.M., Je M.G., Park J.H., Ben H., Koh K.H., Jeon I.-H. (2025). Comparative clinical outcomes and patient satisfaction in primary vs. revision total elbow arthroplasty. J Shoulder Elbow Surg.

[19] Savvidou O.D., Zampeli F., Koutsouradis P., Chloros G.D., Kaspiris A., Sourmelis S. (2018). Complications of open reduction and internal fixation of distal humerus fractures. EFORT Open Rev.

[20] Schoch B.S., Werthel J.D., Sánchez-Sotelo J., Morrey B.F., Morrey M. (2017). Total elbow arthroplasty for primary osteoarthritis. J Shoulder Elbow Surg.

[21] Schwartz J.M., Ramamurti P., Werner B.C., Dacus A.R. (2024). Does timing of total elbow arthroplasty after distal humerus fracture affect 2-year complication rates? J. Shoulder Elbow Surg.

[22] Seok H.-G., Park J.-J., Park S.-G. (2022). Comparison of the complications, reoperations, and clinical outcomes between open reduction and internal fixation and total elbow arthroplasty for distal humeral fractures in the elderly: a systematic review and meta-analysis. J Clin Med.

[23] Sørensen B.W. (2014). Primary total elbow arthroplasty in complex fractures of the distal humerus. World J Orthop.

[24] Stone A., Chan G., Sinclair L., Phadnis J. (2023). Elbow arthroplasty in trauma-current concepts review. J Orthop.

[25] Tarallo L., Celli A., Delvecchio M., Costabile L., Ciacca G., Porcellini G. (2024). Long-term outcomes and trends in elbow arthroplasty with Coonrad-Morrey prosthesis: a retrospective study in large group of patients. Int Orthop.

[26] Viveen J., van den Bekerom M.P.J., Doornberg J.N., Hatton A., Page R., Koenraadt K.L.M. (2019). Use and outcome of 1,220 primary total elbow arthroplasties from the Australian Orthopaedic Association National Joint Arthroplasty Replacement Registry 2008–2018. Acta Orthop.

[27] Yetter T.R., Weatherby P.J., Somerson J.S. (2021). Complications of articular distal humeral fracture fixation: a systematic review and meta-analysis. J Shoulder Elbow Surg.

